# Finite Element Analysis of Electrically Excited Quartz Tuning Fork Devices

**DOI:** 10.3390/s130607156

**Published:** 2013-05-30

**Authors:** Roger Oria, Jorge Otero, Laura González, Luis Botaya, Manuel Carmona, Manel Puig-Vidal

**Affiliations:** Department of Electronics, University of Barcelona, Marti Franques, 1, 08028 Barcelona, Spain; E-Mails: roria@el.ub.es (R.O.); jotero@el.ub.es (J.O.); lgonzalez@el.ub.es (L.G.); lbotaya@el.ub.es (L.B.); mcarmona@el.ub.es (M.C.)

**Keywords:** quartz tuning fork, finite element modeling, piezoelectric sensors

## Abstract

Quartz Tuning Fork (QTF)-based Scanning Probe Microscopy (SPM) is an important field of research. A suitable model for the QTF is important to obtain quantitative measurements with these devices. Analytical models have the limitation of being based on the double cantilever configuration. In this paper, we present an electromechanical finite element model of the QTF electrically excited with two free prongs. The model goes beyond the state-of-the-art of numerical simulations currently found in the literature for this QTF configuration. We present the first numerical analysis of both the electrical and mechanical behavior of QTF devices. Experimental measurements obtained with 10 units of the same model of QTF validate the finite element model with a good agreement.

## 1. Introduction

In the past several decades, Atomic Force Microscopy (AFM) has been employed to measure the nano-scale properties of both soft [1–3] and non-coated [4,5] samples due to the high resolution of the technique. The ability to image, measure, and manipulate matter at the nano -and even atomic scale [6], is the defining attribute which has led to AFM being considered such a valuable and successful tool in nanotechnology. AFM techniques are based on the measurement of the interaction forces between a nanometer-sized tip and the sample surface [7]. Commercial AFM nanosensors are based on the fabrication of microcantilevers with a very sharp tip [8,9]. However, commercial AFMs are limited in their functionality within certain scenarios. High stiffness of the cantilever is required in order to achieve small oscillation amplitudes and to prevent noisy measurements [6], which is not always accomplished. Additionally, the quality factor is often diminished when AFMs are immersed in liquid environments, and they are quite difficult to implement in multi-probe configurations [10].

As an alternative to classical AFM nanosensors, the use of Quartz Tuning Forks (QTFs) has been proposed. QTFs are piezoelectric devices that are commonly known for their application in customer electronics. In 1989, QTFs started to be used in microscopy [11–14]. One great advantage is the ability to combine the functions of a sensor and actuator, thus reducing the overall instrument complexity. In addition, QTF offers a stable and a very narrow band [15]. Moreover, higher quality factors (*Q*) are attributed to QTFs, as opposed to standard cantilevers, which make them suitable for applications in a liquid environment. Different analytical models of QTFs have been developed in the literature [16–21], but the dynamics response of the electrical and mechanical coupling of these devices remains unclear. All of these models are based on the cantilever configuration for mechanical excitation, except in the case described in [19], where an electrical circuit is introduced as a means to drive the QTF to the resonance frequency leading to compensation between the electrical energy and the mechanical energy of the QTF.

Finite Element Analysis (FEA) has been widely used in sensor analysis and design [22–24] as an alternative to the analytical models. So far, only a few studies have been found [18,25–29] with respect to QTFs. In [18,26] a modal analysis and calculation of the static capacitance for an optimum design is reported. In [25] the modal analysis of the sensor is reported. In study [28], the oscillator behavior of the QTF probe for different angles of the attached tip is studied for different vibrational modes. In [27,29] experimental data and numerical simulations are reported to compare and quantify the spring constant of the QTF. The main limitation of these models is that they do not consider the electromechanical coupling for the dynamic response of the device. In order to obtain accurate results, the complete system must be analyzed carefully; otherwise, uncertainties such as material properties, dimensions of the sensor—especially separation between the prongs and the base contribution of the QTF—and the electrode definition, which can introduce shifts in parasitic contribution, will likely lead to erroneous results.

We present herein a 3D finite-element model of the QTF, which models the coupling between the mechanical and the electrical behavior of the device by implementing the electric part, composed of a: voltage source, electrodes, and compensation circuit. In the first section, the mechanical model is presented. In the second section, the electromechanical model highlights the importance of accurately defining the geometry of the two electrodes. Finally, experimental measurements, which validate our finite element model, are shown.

## 2. Sensor Principles and Modeling

### 2.1. Theory

The tuning fork is a bimorph cantilever based on the piezoelectric properties of the quartz. The sensor consists of two prongs attached to a base, which normally is clamped to a holder. Each of the prongs is coated with a thin layer of a conductor material, which permits the resonator to be driven either electrically or mechanically. In the former case, a potential difference between the electrodes is applied, whereas in the second one a dither is attached to one of the prongs of the QTF. Given the type of driver employed, either the electrical current through the device—for electrical excitation—or the generated piezoelectric voltage—for mechanical excitation—are related to the fork's vibration amplitude.

In microscopy applications, the QTF is driven to its resonance frequency by either mechanical or electrical excitation. There are two main vibrational modes for the same mechanical deformation: in-phase and anti-phase modes. For electrical excitation, the generated current can just be measured in the anti-phase mode because the current is only generated in the system when both prongs are vibrating in opposite directions.

In order to properly characterize the QTF dynamics, analytical models have been proposed in the literature [16–21]. Models from [16,19] have succeeded compared to the others by establishing a now well-known formulation for the calculation of those parameters, which are not straightforward to obtain. The main two parameters that these models propose are the spring constant (*K*) and the amplitude of oscillation (*A*) of the sensor. The spring constant of the sensor can be expressed as [19]:

(1) $$K=0.2575\frac{{\text{TW}}^{3}E}{L^{3}}$$

where *T*, *W* and *L* correspond to the dimensions of the thickness, width, and length of the prongs of the QTF, respectively; and *E* is the Young's modulus of the quartz.

It is worthwhile to mention that the calculation of the spring constant has led to great controversies and discrepancies in the research community due to the lack of a generalized model. Although the cantilever-based model for studying the dynamics of QTF is the most accepted model, a two-coupled oscillator model has been utilized in [17,20,21]. The coupled-oscillator model discusses the necessity to link the dynamics of the two prongs of the QTF. In [20,21] it is reported that the calculation of the spring constant using the cantilever model underestimates its true value. In contrast, [27] it shows that the cantilever model overestimates the spring constant value. Moreover, in studies [27,28] the contribution of the QTF base in the calculation of the spring constant is discussed. The differences seen in the literature [13,21,27], can play a crucial role if quantitative measurements are needed.

In the case of electrical excitation, the amplitude of oscillation can be calculated by using the following [19]:

(2) $$A=\sqrt{\frac{V_{\text{rms}}I_{\text{rms}}Q}{K2\pi f_{0}}}$$

where *V_rms_* is the amplitude of the excitation signal, *I_rms_* the measured current, and the quality factor, defined as:

(3) $$Q=\frac{f_{o}}{\Delta f}$$

where *f_0_* is the resonance frequency and *Δf* is the bandwidth of the QTF when the amplitude decays 3 dB.

### 2.2. Model of the Mechanical Structure Using Finite Element Model

**Figure 1.**
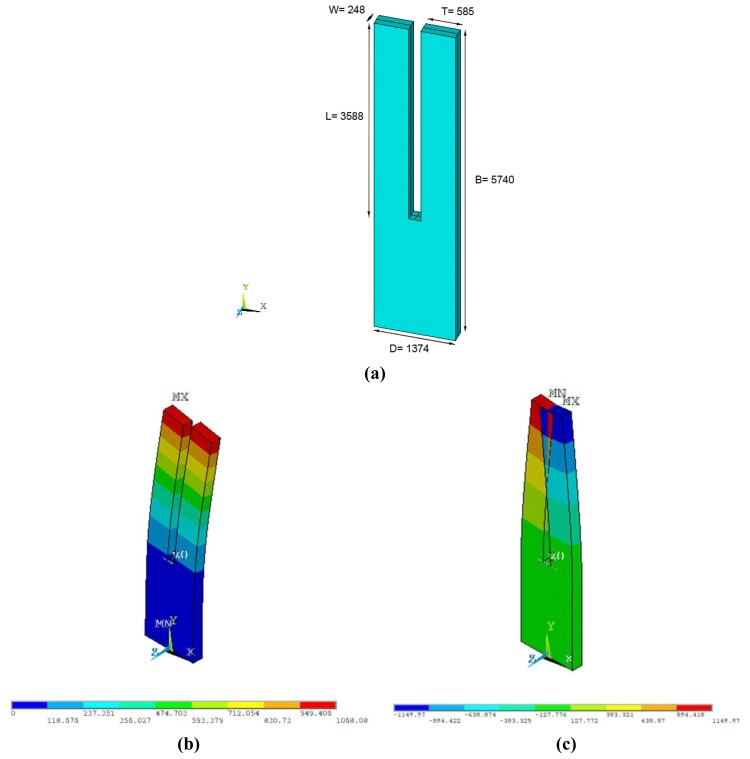
(**a**) Dimensions and geometry of QTF (in μm) (**b** and **c**) Vibrational modes for in-phase and anti-phase mode of QTF. Bar colors indicate arbitrary units of the displacements along the X coordinate.

In this work, a finite element model (FEM) of the QTF is proposed. In order to make an accurate 3D model, dimensions of the QTF are set in accordance to measurements carried out in an optical microscope (B-353MET model, Euro-Microscopes). In contrast to commercial AFM sensors where cantilevers present a rotation with respect to the X coordinate, the tuning fork model rotates with respect to the Z coordinate; thus, the width (*W*) and the thickness (*T*) are defined in a opposite ways compared to AFM cantilevers, as seen in Figure 1.

As it was previously stated, the QTF is based on piezoelectric phenomena. Therefore, linear piezoelectricity equations of elasticity have to be defined and coupled to the electrostatic charge by means of piezoelectric constants [30]:

(4) $$\begin{array}{ccc} \left[\begin{array}{c} \epsilon_{p}\\ D_{i} \end{array}\right] & =\left[\begin{array}{cc} S_{\text{pq}}^{E} & d_{\text{pk}}\\ d_{\text{iq}} & \epsilon_{\text{ik}}^{\sigma } \end{array}\right] & \left[\begin{array}{c} \sigma_{q}\\ E_{k} \end{array}\right] \end{array}$$

(5) $$\begin{array}{lll} \left[\begin{array}{l} \sigma_{p}\\ D_{i} \end{array}\right] & =\left[\begin{array}{ll} Y_{\text{pq}}^{E} & e_{\text{pq}}\\ e_{\text{iq}} & \epsilon_{\text{ik}}^{\epsilon } \end{array}\right] & \left[\begin{array}{l} \epsilon_{q}\\ E_{k} \end{array}\right] \end{array}$$

{*σ_p_*}=Stress vector
{*D_i_*}=Electric flux density vector
{*E_K_*}=Elastic strain vector
{*∈_q_*}=Electric field intensity vector
$[Y_{\text{pq}}^{E}]=\text{Elasticity matrix}$
[*e_pk_*]=Piezoelectric stress matrix
$[\epsilon_{\text{ik}}^{\epsilon }]=\text{Dielectric matrix}$

The different physical properties of quartz have been widely studied [31,32]. However, elastic, piezoelectric, and the dielectric permittivity matrices differ slightly among the various reported results. In the present work, properties from [32] have been chosen because more sophisticated measurement techniques—based on resonance ultrasound spectroscopy—are used to determine the values. The piezoelectric coefficients can be seen as follows:

$$\left[Y_{\text{pq}}^{E}\right]=\left[\begin{array}{cccccc} 86.76 & 6.86 & 11.85 & -18.02 & 0 & 0\\ 6.86 & 86.76 & 11.85 & -18.02 & 0 & 0\\ 11.85 & 11.85 & 105.46 & 0 & 0 & 0\\ -18.02 & 18.02 & 0 & 58.14 & 0 & 0\\ 0 & 0 & 0 & 0 & 58.14 & -18.02\\ 0 & 0 & 0 & 0 & 18.02 & 39.95 \end{array}\right]\text{GPa}$$

The piezoelectric constant matrix, which permits the structural and electrical behavior of the material to be coupled, is defined as follows:

$$\left[e_{\text{pk}}\right]=\left[\begin{array}{ccc} -0.151 & 0 & 0\\ 0.151 & 0 & 0\\ 0 & 0 & 0\\ 0.061 & 0 & 0\\ 0 & -0.061 & 0\\ 0 & 0.151 & 0 \end{array}\right]\frac{C}{m^{2}}$$

The piezoelectric behavior of the material is accomplished by using the element type SOLID226 in ANSYS.

The model is defined in uMKS units according to ANSYS nomenclature. The model is composed of 12,384 hexahedral elements with a size of 60 μm, which is translated into 59,340 nodes. In addition, the bottom of the base is constrained to emulate the clamped structure of the real device.

### 2.3. Electrodes Definition and Parasitic Capacitance Compensation

For the implementation of the electrical part and to couple it with the mechanical behavior of the QTF, the loaded and the grounded electrodes needed to be defined. In addition, the electrodes also define the way in which the deformation occurs when an electric field is applied, and hence the type of acoustic wave generated [33].

**Figure 2.**
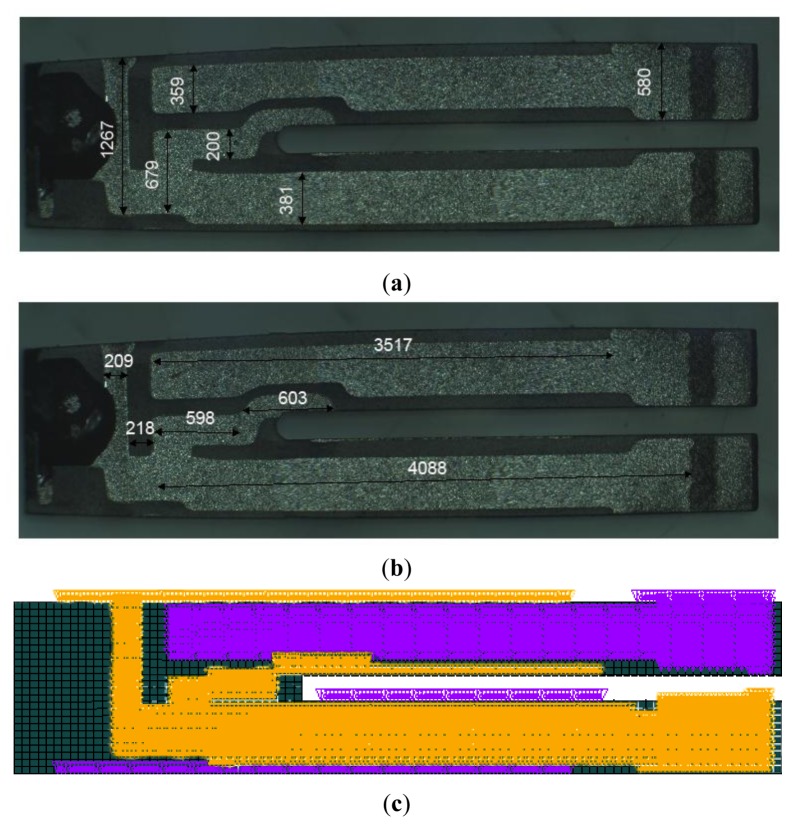
(**a** and **b**) Electrodes of the QTF are according to the optical microscope measurements. (**c**) The electrodes in ANSYS are defined at the bottom and top face of the QTF symmetrically. Dimensions are in μm.

As shown in Figure 2(a,b), the electrodes are placed in this particular alignment to obtain and optimize the maximum charge transfer. The electrodes were defined on the surface nodes of the tuning fork according to optical microscope measurements, thus simulating the thin layer of the conductor material. The surface nodes of each electrode of the QTF are coupled together, which sets the same amount of voltage for each node.

Therefore, two groups of nodes are created. Each electrode is defined by 2,317 elements that are translated into 6,390 nodes. An independent voltage source links one node from each of the two groups together implemented by using the element CIRCU94. One group is defined as the loaded electrode such that a symmetric sinusoidal voltage wave is supplied to it, while the other electrode is grounded.

**Figure 3.**
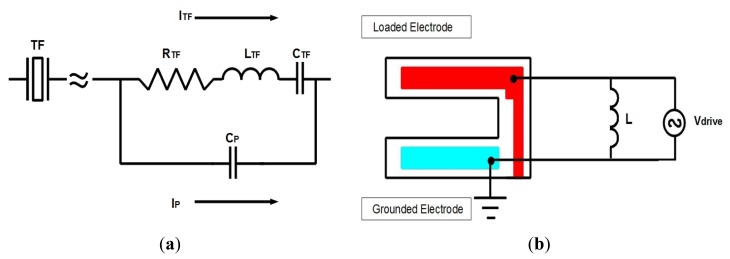
(**a**) Equivalent circuit of the QTF (Butterworth-Van Dyke model) (**b**) Electric circuit composed of power supply and inductor. The power supply permits the QTF to be driven electrically, and the inductor allows compensating for the parasitic capacitance.

The electrical part of the QTF is modeled by an equivalent circuit (Butterworth-Van Dyke model) based on a serial RLC circuit with a parallel capacitor [34]. The resistor models the dissipation, the capacitor and the inductor model the potential and the kinetic energy storage, and the parallel capacitor models all the parasitic contributions due to contacts, cables, *etc.* (Figure 3(a)).

One of the main problems is that the current flowing through the QTF becomes dominant away from the resonance frequency due to parasitic capacitance; this phenomenon is responsible for asymmetries and shifts in the frequency response. Thus, it is required to compensate for the parasitic current. In the proposed model, an inductor element with CIRCU94 is implemented within ANSYS for this very reason, whereas a means to compensate in the experimental setup will be explained later. Therefore, the voltage source and the inductor determine the final excitation circuit.

Parasitic capacitance can be obtained by doing harmonic simulation over a broad frequency range by interpreting the contribution of this capacitance as the slope of a linear fit dependent on the frequency response. Using Equation (8), the value of the inductor is modeled from the real part of the impedance equal to the parasitic capacitance:

(6) $$X_{c}=X_{L}$$

(7) $$\frac{1}{2\pi \;f\;c}=2\pi \;f\;L$$

(8) $$L=\frac{1}{{\left(2\pi \;f\right)}^{2}C}$$

In order to perform the harmonic simulation, one more input parameter is needed: the quality factor of the QTF. The quality factor is defined in numerical simulations through the damping ratio (*ξ*) of the device:

(9) $$\xi =\frac{1}{2Q}$$

For our model, the inductance was calculated to be 26.5 H by using Equation (8).

Parasitic contribution in the finite element model is only due to the electrodes rather than contacts, cables, *etc*. Therefore, the model is expected to have less parasitic capacitance than in the experimental setup. However, one must keep in mind that this compensation plays an important role in the experimental configuration of the QTF probe.

Concerning the way in which the measurements are taken, the device is electrically driven, and the amplitude of oscillation is obtained by measuring the current through the QTF. An AC voltage source at the resonant frequency of the fork is applied, and the current is measured by a transimpedance amplifier (TIA) with a gain R_G_ = 10^6^ (V/A).

**Figure 4.**
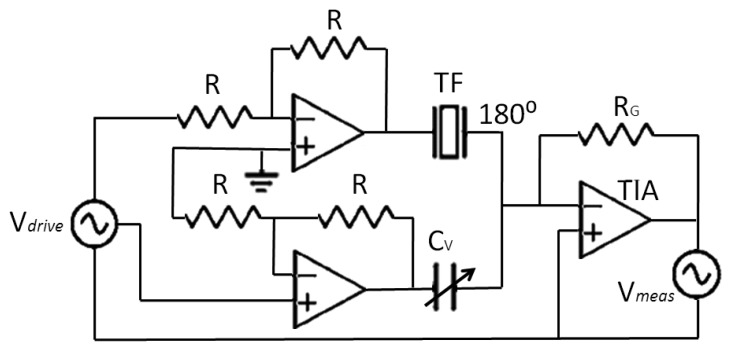
Implemented circuit for parasitic capacitor compensation.

In the experimental setup, a capacitor-compensated circuit was implemented to drive the QTF [35]. This type of compensation circuit was chosen because of the high magnitude of inductor, which would make it infeasible to implement practically. In that circuit (Figure 4), the parasitic current is compensated with a sub-circuit with the same capacitance by using a tunable variable capacitor, but with 180° phase.

As a consequence, only the current through the nanosensor is amplified by the TIA, and it is translated into voltage to measure the extent of oscillation with a lock-in amplifier [15].

## 3. Results and Discussion

The first objective in validating our model was to obtain the resonance frequency of the QTF, for both the in-phase and anti-phase vibrational modes.

For the in-phase mode, when both prongs are vibrating in the same direction, our model demonstrates a resonance frequency of 27,433 Hz. However, we have not verified this value experimentally due to the small readout signal that is not distinguishable from the intrinsic noise of the equipment. Nevertheless, in study [21] the in-phase mode shows significant agreement with our model.

Regarding the anti-phase mode, when both prongs are vibrating in the opposite direction, the resonance frequency is completely observed and well correlated with the nominal value of 32,768 Hz provided by the manufacturer [36]. Table 1 summarizes the different vibrational modes seen below:

In order to validate the dynamics response of the model proposed, ten QTFs of the same model and manufacturer (*Abracon* AB38T) were electrically driven from 10 mV up to 100 mV. The quality factor and the current through the devices were measured. In Table 2 the quality factor of the ten devices, and the respective damping ratios, which are used in the simulations are shown. *Q* factors of the sensors were calculated by applying a pseudo-Lorentzian fit [15] to the experimental current *versus* frequency curve [16].

**Figure 5.**
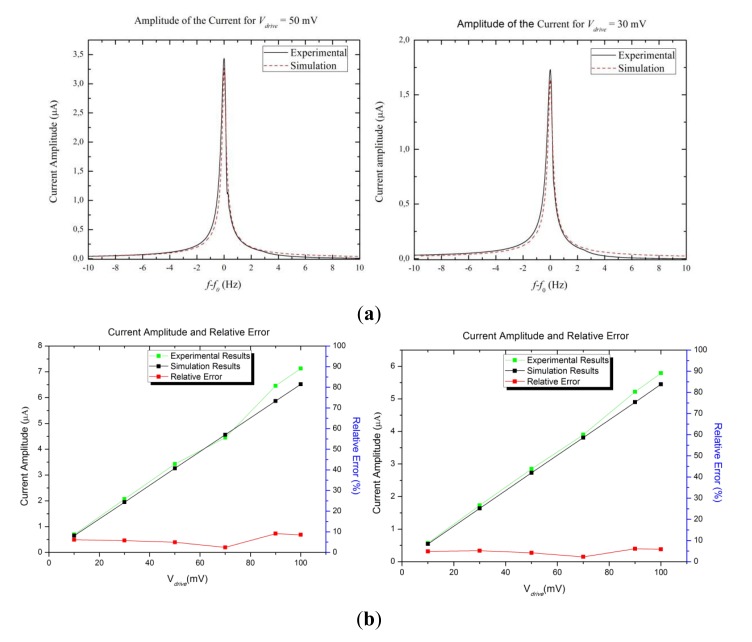
(**a**) Amplitude of the current for two QTFs for *V_drive_* 30 mV and 50 mV, respectively. (**b**) Current peaks at the resonance frequency and relative error between experimental and numerical data.

The first step to validate the model was to compare experimental and simulation data for one tuning fork with several *V_drive_*. As shown in Figure 5, the amplitude of the current is proportional to the *V_drive_* as expected. The agreement of the model for all ten devices is between the 91% and 98%.

In order to properly validate the proposed model, several QTFs have to be characterized. Comparison of the ten different QTFs was performed between measured and simulation data for a single *V_drive_*.

The amplitude of oscillation can be experimentally obtained by interferometric techniques [15,37], which are difficult to practically carry out, or theoretically obtained from Equation (1). This parameter is necessary to fully understanding the electromechanical behavior of the QTF; our model presents a straightforward approach.

**Figure 6.**
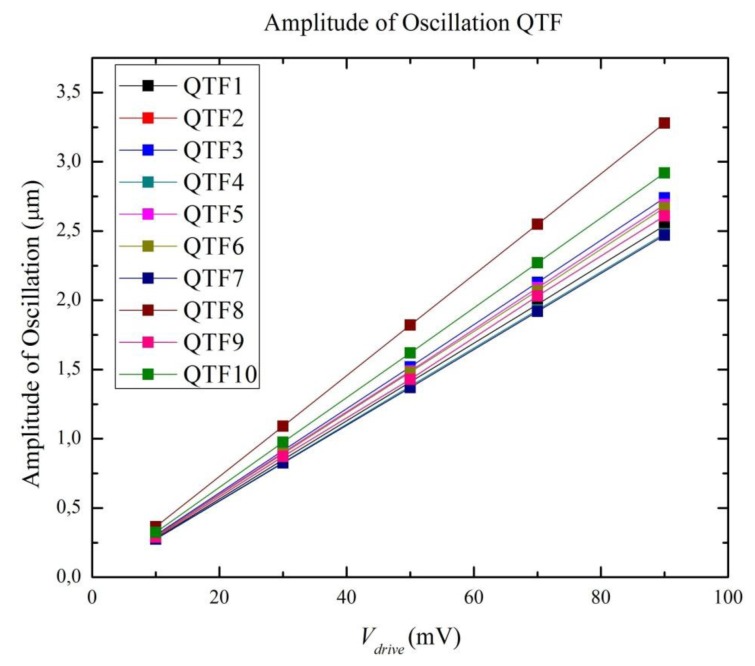
Results of the amplitude of oscillation of the ten QTFs from the finite element model.

As shown in Figure 6, simulation data indicates a linear relationship, which may be attributed to the fact that harmonic simulations in ANSYS resolve the time-dependent equations of motion for linear structures undergoing steady-state vibration. However, this confirms the actual electromechanical behavior of the QTF, which had been previously identified by examining the electrical current peaks through the QTF for the chosen range of *V_drive_*. Therefore, it can be assured that the QTF behaves linearly between 10 mV and 100 mV, which is well captured by the proposed model.

**Figure 7.**
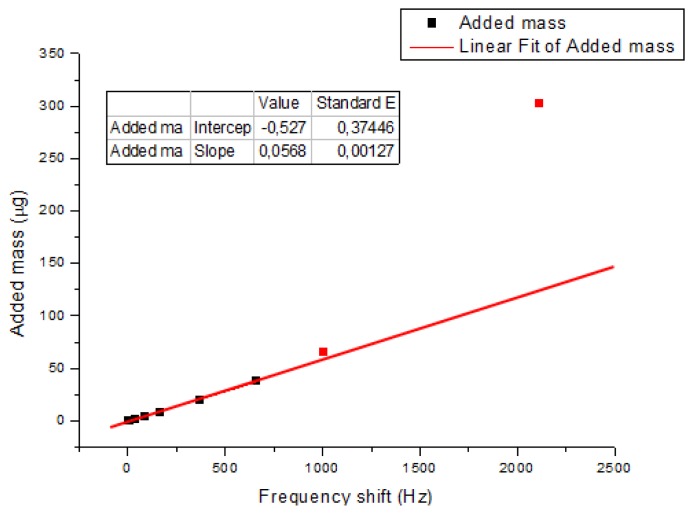
Results of the frequency shift produced by the added mass from the finite element model.

Finally the validation of the QTF as a sensor was done by small-mass loading one of the prongs of the device in the finite element model and measuring the shift in the resonant frequency produced by the added mass. A small cube of solid material with a known mass was coupled to the left prong of the QTF, in a similar way that the experiments conducted in [21] and [38]. Results are shown in Figure 7. The sensitivity of the device as mass sensor was 57 ng/Hz, which was in good agreement with results reported for the experimental measurements in [38]. For high added masses, the model shows a non-linear behavior, as shown in the experiments in [21].

## 4. Conclusions

A new model of quartz tuning fork with two free prongs and electrically excited is presented based on finite element analysis developed in ANSYS by incorporating the electrical part: excitation, compensation circuit and the electrodes. The model has been validated by measuring ten separate quartz tuning forks at different driving amplitudes—from 10 mV to 100 mV—which exhibit a strong agreement between 91% and 98%. The remaining error can be attributed to small geometric differences between the electrodes of the model and the electrodes from the actual quartz tuning fork. In addition, the parasitic capacitance cannot always be completely compensated in the experimental measurements.

Finally, the model proposed herein allows from the comparison between experimental and simulation data, which is complicated to achieve from other models in the literature. These analytical models are normally developed for mechanically excited quartz tuning forks implying that the dither driving energy must be determined. However, this is no easy task due to the appearance of mechanical losses and the coupling between the sensor and dither, which are difficult to quantify. Our developed model also overcomes the difficulty of measuring certain parameters, such as the amplitude of oscillation and the sensitivity. With the results obtained, the model could be used to calculate the effective spring constant of the device as deeply discussed in [21].

## Figures and Tables

**Table 1.** Resonance frequency for the in-phase and anti-phase vibrational modes.

	**In-Phase Mode [Hz]**	**Anti-Phase Mode [Hz]**
Castellanos *et al.* [21]	27,800	32,766
Experimental Results	-	32,768
FEM Results	27,433	32,768
Manufacturer [36]	-	32,768

**Table 2.** Quality factor of the ten QTF. *Q* values were experimentally measured, and implemented in FEM through the damping ratio.

**QTF**	**Quality Factor (*Q*)**	**Damping Ratio (ξ)**
QTF1	104542	4.78e-06
QTF2	112800	4.43e-06
QTF3	113077	4.42e-06
QTF4	102144	4.89e-06
QTF5	110724	4.51e-06
QTF6	109778	4.55e-06
QTF7	101471	4.92e-06
QTF8	135054	3.70e-06
QTF9	107489	4.65e-06
QTF10	120019	4.16e-06
